# Hints for the Sick-Room

**Published:** 1888-08-04

**Authors:** M. M. Basil

**Affiliations:** Author of "The Commoner Accidents to Life and Limb."


### HINTS FOR THE SICK-ROOM.

By M. M. Basil, M.A., M.B., C.M., Author of "The Commoner Accidents to Life and Limb."

ADMINISTRATION OF MEDICINES (*continued*).

CONSIDERABLE amount of tact is necessary to get some patients to take medicine. Generally the mouth, but not the lips, should be moistened previously. Oily medicines should not touch the lips. The persistent taste of some strongly-bitter drugs—quassia, quinine, strychnine—is best removed by giving the patient some bread to chew and swallow repeatedly in small quantities. This wipes mechanically the back of the tongue, and removes the bitter taste which is perceived mainly in this situation. Many of the nauseous medicines really offend by their smell and not by their taste; in such cases, if the nostrils be closed with the fingers before the medicine comes within sight and smell, and kept closed until the mouth is washed out subsequent to taking the draught, hardly any taste will be perceived by the patient. Some people are extremely awkward in taking m dicines but it is not rare to find that their attendants are equal awkward in their methods of persuasion. Holding a draught containing valerian or assafœtida right under the nose of the patient while coaxing her to take it, is not the best road to success. Some medicines are difficult to swallow if not well diluted. Preparations containing sulphuric ether or spirits of ammonia come under this category. The sensation of choking produced by them in their concentrated condition is due to their local action, and can be done away with by free dilution.

Medicines may be given before, after, or between meals, according to circumstances. Some drugs have no special relationship with the meal hours. In each case you should distinctly ascertain the time when medicine should be administered. Some of the general principles which may guide you are: Medicines intended to act not locally on the stomach but constitutionally on the body, are given after meals; such are preparations of iron, arsenic, zinc, quinine, &c. Acids and alkalies may be given before or after meals, according to the effect expected of them. Commonly, however, alkalies are given before meals, acids soon after meals. When nothing is expressed to the contrary, you will not be far wrong in giving medicines at the middle of the interval between two meals. When two or more medicines are to be given to the patient, you should so arrange the programme as to keep their times of administration as wide apart as possible. Cod liver oil may be given immediately before or after a meal, an hour after it, or only at bedtime.

Purgative mixtures or powders are best given early in the morning. The saline purgatives must be well diluted. To effect this it is sufficient to give the dose in a small quantity of water, and then administer more water to wash it down.

If mixtures containing acids or preparations of iron are to be administered for some time, it is best to allow the patient to take them through a glass tube or a quill, so as not to bring them freely in contact with the teeth. A weak lotion of baking soda is useful in washing out the mouth after taking acids. You must be particularly careful when sulphuric acid is used, since it blackens the teeth badly. Remember this please, because should you blacken a patient's teeth by oversight, you may feel certain that your character for nursing will be blackened in return, and that not by any oversight.

Medicines given to produce vomiting should be followed up by plenty of warm water. You should be quite ready here for certain speedy results, for, as the Irishman said, "Emetics are awful things to keep down on the stomach." It would also be advisable to be ready to meet promptly analogous accidents in cases of perineal or vesical examinations and operations.

The prevention of vomiting subsequent to taking medicines, not intended to act as emetics, deserves careful consideration. The nauseous taste must be removed from the mouth as speedy as possible. The patient must be kept in the recumbent position, by preference on the left side, and no fluids allowed after the medicine. If vomiting has occurred on previous occasions, no liquids must be allowed for a couple of hours before the administration of the drug.

It would be well for you to avoid exposing yourselves to an accident which is rather embarrassing to all interested in the treatment of the sick. It may happen that a nervous or suspicious patient is fussy about taking a mixture, because, she asserts, it is given to poison her. Do not swallow the draught yourself to prove her suspicions groundless. For, should it by any possibility make you sick, the patient's absurd fancies become facts proved to demonstration. Constant work by the sick bed, want of out-door exercise, night watching, and other causes combine to render nurses and doctors extremely bad subjects for trying test experiments upon. You should, therefore, carefully decline making such trials on yourselves. The good faith of doctors and nurses can be defended when necessary by other means than the primitive one of swallowing a suspected draught.

You should give no information to the patient as to the nature of the medicine prescribed for her. It is due to the doctor responsible for the case that any legitimate inquiry on this point be directly addressed to him. Information, however innocent in appearance, may be abused in every conceivable way to the detriment of the patient. If opium or morphia is being administered by any method, it would be almost a breach of confidence to communicate the information directly to the patient. There appears to be no doubt that a few patients have contracted the habit of opium-eating by the medicinal use of the drug. Your own sense of responsibility will decide it for you whether it is right to communicate to a patient information of a kind which can lead to no possible good, and is by no means devoid of grave risks.

All powerful medicines should be kept under lock and key. One of the most dangerous of these agents is carbolic acid. Strong lotions, liniments, ointments, acids, and alkalies require careful attention. Sleeping draughts, bottles concontaining chloroform or opium must not be left within reach of patients, especially of those who suffer from prolonged wakefulness. In the distracted mental condition which is common to such, a fatal dose may be taken during your absence, or in the night.

If the patient is permitted to use a lotion or liniment in an ordinary cup or saucer, you must be certain that there is no possibility of a fatal blunder occurring to himself or others. Is he not likely to mistake it for tea, and drink it during his sleepy moments? Liniments that contain opium or spirits must be kept out of the reach of opium eaters and drunkards. The opium eater will stick at nothing in order to gratify his morbid craving. Liniments must not be warmed over a flame, as that of a spirit lamp, else a sudden conflagration on a small scale may be the result. They may be warmed easily in a saucer over some hot water.

Some degree of discretion must be used in obeying orders which are expressed in doubtful words. If you are absolutely ignorant of the nature of the drug to be administered, it is necessary to have all doubtful points in the instructions cleared up before obeying them. In other circumstances the spirit of the instructions must be carried out, not their letter. "A pill or draught every hour or couple of hours until the patient sleeps," must not be strained too far. The patient may not sleep till he gets enough to make him sleep too long.

*(To be continued.)*

